# Cardiac Conduction System as an OAR in Radiation Therapy: Doses to SA/AV Nodes and Their Reduction

**DOI:** 10.1016/j.ijpt.2024.100631

**Published:** 2024-09-26

**Authors:** Martin Domanský, Michal Andrlík, Samuel Kurucz, Jan Vilimovský, Sarah Al-Hamami Salih, Daniela Šimánková, Jiří Kubeš

**Affiliations:** 1 Proton Therapy Center Czech, Prague, Czech Republic; 2 Department of Oncology, 2nd Faculty of Medicine, Charles University Prague and Motol University Hospital, Prague, Czech Republic; 3 Institute of Nuclear Medicine, First Medical Faculty Charles University, General University Hospital Prague, Prague, Czech Republic

**Keywords:** Cardiac substructure, Cardiotoxicity, Proton therapy, Non–small cell lung cancer, Conduction nodes

## Abstract

**Purpose:**

Despite advancements in radiation techniques, concerns persist regarding the adverse effects of radiation therapy, particularly cardiotoxicity or radiation-induced heart disease. Recently, arrhythmogenic toxicity has come to the forefront—the impact of radiation therapy on the cardiac conduction system. Our objective was to conduct a dosimetric study and subsequently investigate the feasibility of optimizing the sinoatrial (SA) and atrioventricular (AV) nodes as organs at risk (OARs) in proton radiation therapy for non–small cell lung cancer with N3 disease.

**Patients and Methods:**

Thirty-two non–small cell lung cancer patients with N3 disease undergoing proton radiation therapy were included. Sinoatrial and AV nodes, along with standard OARs, were delineated. Dosimetric analysis and optimization were performed using intensity-modulated proton therapy.

**Results:**

Patients surpassing a predefined SA node dose threshold underwent dose optimization. Proton radiation therapy with pencil beam scanning demonstrated a significant reduction in SA and AV node doses without compromising target volume coverage or significant shift in the dose to other monitored OARs.

**Conclusion:**

Dose reduction to the SA and AV nodes for pencil beam scanning is a relatively simple task, and the reduction can be very substantial. Larger cohort studies and diverse radiotherapeutic modalities are needed for further validation and refinement of dose constraints.

## Introduction

Despite significant advances in radiation techniques, the adverse effects of radiation therapy remain a major concern in contemporary radiation oncology. Radiation toxicity to the heart, that is, cardiotoxicity or radiation-induced heart disease, occurs in patients undergoing radiation therapy in the thoracic region. Historically, cardiotoxicity has been primarily perceived as radiation-induced coronary artery disease, structural myocardial damage, and valvular dysfunction. Cardiovascular diseases resulting from these phenomena represent a leading cause of death among patients who have undergone radiation therapy in the heart region.^1^ Definition, diagnosis, treatment, and management of these conditions are addressed by guidelines on cardio-oncology, first published in 2022 by the European Society of Cardiology.^2^ However, radiation affects all tissues, leading to a highly heterogeneous manifestation of radiation-induced heart disease. Recently, arrhythmogenic toxicity has come to the forefront—the impact of radiation therapy on the cardiac conduction system. Following radiation therapy, a spectrum of conduction disorders may occur, including supraventricular arrhythmias (atrial fibrillation, flutter), ventricular arrhythmias (ventricular tachycardia, extrasystoles), bradyarrhythmias, blocks, and tachyarrhythmias.^1^, ^3^, ^4^ Many of these arrhythmias are potentially life-threatening, contributing to increased morbidity, mortality,^5^, ^6^, ^7^, ^8^, ^9^, ^10^, ^11^ or reduced quality of life, for example, due to the necessity of pacemaker implantation.^11^, ^12^

Sinoatrial (SA) and atrioventricular (AV) nodes are not currently routinely monitored as organs at risk (OARs). However, dosimetric studies on the conduction system^13^, ^14^, ^15^, ^16^ and research exploring the influence of radiation dose on the conduction nodes concerning arrhythmogenic toxicity or increased mortality and morbidity have begun to emerge.^10^, ^11^ The primary objective of our study was firstly to supplement limited dosimetric data and studies related to the conduction structures of the heart (specifically SA and AV nodes) and secondly to verify the feasibility of sparing these structures through proton radiation therapy after their inclusion as OARs. In this study, we selected patients with non–small cell lung cancer (NSCLC) with N3 disease, in whom, in our judgment, cardiac structures (including conduction nodes) would be exposed to a high dose.

## Methods and materials

### Study population

A total of 32 patients with NSCLC treated from September 2016 to March 2023 were included in this dosimetric study. These patients had N3 disease, which indicates metastases in the contralateral mediastinal, contralateral hilar, ipsilateral or contralateral scalene, or supraclavicular lymph nodes. Contouring for these cases is based on delineating positron emission tomography-positive findings (primary tumor and affected nodes). According to internal methodology, selected elective areas with a high risk are also delineated. These patients have large target volumes, leading to an anticipated high dose to the heart and its structures. They were in clinical stages IIIB-IV. In this sample, 20 patients (62.5%) had a right-sided tumor, 9 had a left-sided tumor (28%), and 3 had an indistinguishable/bilateral location (9%). Radiation was delivered either in 2 phases with a gradual reduction of target volumes or using the technique of simultaneous integrated boost, with a total dose ranging from 60 to 75 GyE (Gray equivalent)/25 to 30 fractions for the primary tumor and 50 to 54 GyE/25 to 30 fractions for elective lymphatic areas.

### Cardiac structures delineation and dosimetry

**Figure 1:**
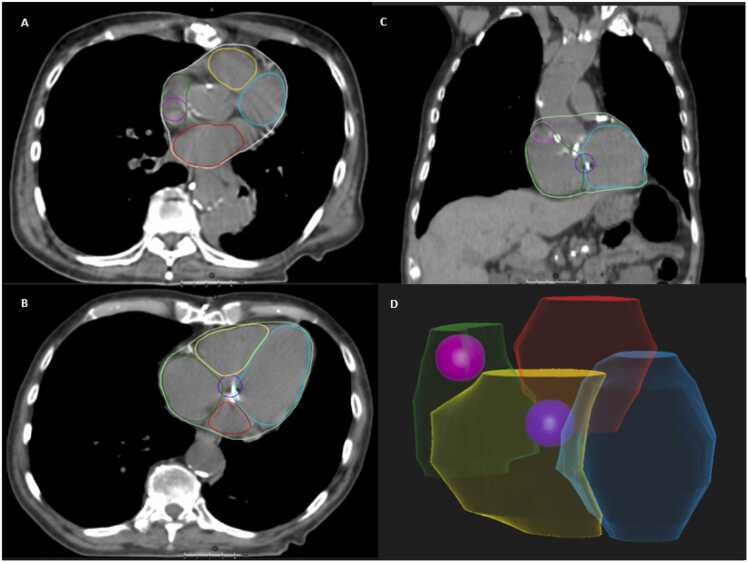
Contouring of SA and AV nodes. Description: pink—SA node, purple—AV node, blue—left ventricle, yellow—right ventricle, green—right atrium, and red—left atrium. (A and B) Axial images, (C) frontal image, and (D) 3-dimensional representation. Abbreviations: AV, atrioventricular; SA, sinoatrial.

Source: Domanský Martin (2023), RayStation Clinical System (12A SP1), Proton Therapy Center Czech, Prague.

SA and AV nodes were contoured for patients in addition to the originally standard monitored structures (atria, ventricles, coronary arteries, whole heart, lungs, spinal cord, esophagus, trachea, thyroid gland). Planning was conducted using RayStation planning software (Ray Search, Sweden, treatment planning system). First contouring atlas for SA and AV nodes was published by Loap et al.^17^ This contouring technique has been utilized in other dosimetric studies,^13^, ^14^, ^15^, ^16^, ^18^, ^19^ and therefore, it was applied in our study (Figure 1). Sinoatrial node was defined as a sphere with a radius of 10 mm, positioned at the junction of the superior vena cava and the auricula of the right atrium, ensuring it did not extend beyond the entire heart volume. Vertically, the center of the SA node was defined as the point where the aorta fully separated from the left ventricle. The AV node is located 1 cm above the first slice where the left atrium ends. The AV node was defined as a sphere with a radius of 10 mm centered at the junction of the 4 cardiac chambers. Subsequently, the dose received by both nodes during the administered radiation therapy was calculated.

### Optimization process

After analyzing doses to the SA and AV nodes, patients with D2% (dose received by 2% of the volume, ie, maximal dose D_max_) to the SA node exceeding a dose of 5 GyE were selected. This limit was established based on the International Lymphoma Radiation Oncology Group recommendations, which state to minimize cardiac substructure doses as much as possible, ideally under 5 Gy. Out of the original sample of 32 patients, this threshold was surpassed in 23 patients. Doses to the AV node were not considered, as in most cases, they approached 0 or reached low values (see Table 1). For this subset of selected patients with D2% ≥5 GyE to the SA node, dose optimization of the radiation plan was conducted to reduce the dose to the SA node while preserving coverage of target volumes and maintaining dose constraints to OARs.

**Table 1:** Dosimetry sum.

	SA node D2% (ie, D_max_)	SA node D_mean_	AV node D2% (ie, D_max_)	AV node D_mean_
Average value (all)	27.9	14.2	2.0	0.8
Median (all)	26.5	8.6	0.6	0.3
Average value (left laterality)	17.1	6.3	0.6	0.3
Average value (right laterality)	32.5	17.7	2.6	1.0
Median (left laterality)	16.6	6.1	0.4	0.2
Median (right laterality)	29.9	10.2	0.9	0.3
MAX	70.7	51.4	17.3	6.5
MIN	6.3	1.8	0.1	0.0

Abbreviations: SA, sinoatrial; D2%, dose received by 2% of the volume, that is, maximal dose (D_max_); D_mean_, mean dose; AV, atrioventricular; MAX, maximal value; and MIN, minimal value.

Values in Gray equivalent: 1 GyE represents the physical dose of protons multiplied by a relative biological effectiveness factor 1.1 and should thus have similar biologic effects as 1 Gy of photon dose.

All clinically used plans were calculated using RayStation. For older plans to have the best comparative value possible, those that were created using older decommissioned beam mode were reoptimized for the new beam model.

A full intensity-modulated proton therapy (IMPT) optimization was performed. New optimization was carried out, taking into account the SA and AV nodes for these existing treatment plans. Our goal was to achieve the lowest possible dose to the SA and AV nodes without significantly compromising dose coverage to the planning target volume (PTV), clinical target volume (CTV), and gross tumor volume (GTV) or substantially increasing the dose to other OARs (see Table 2). The dose in 2% of the SA and AV node volumes was evaluated (D2%). Assessment criteria included D95% (dose in 95% of the evaluated volume) for PTV and D98% for CTV and GTV. The impact on the lungs, including D_mean_, V_5GyE_ (volume in % receiving more than 5 GyE), and V_20GyE_, was also evaluated, as well as the effect of optimization on the heart V_15GyE_ and D_mean_. The effect of SA and AV node optimization on individual compartments of the heart D_mean_ and esophagus D_mean_ was also evaluated. The dose values were given in Gray equivalent, where 1 GyE represents the physical dose of protons multiplied by a relative biological effectiveness factor of 1.1 and should thus have similar biologic effects as 1 Gy of photon dose.

**Table 2:** Optimization evaluation.

	Average	Min	Max	Median
SA D2_%_ Δ_relative_	62.21%	−87.21%	−17.49%	−65.46%
Original (GyE)	27.93	13.98	6.29	31.47
SA+AV optimized (GyE)	10.55	1.78	5.19	10.87
AV D2_%_ Δ_relative_	−61.33%	−84.00%	64.81%	−38.01%
Original (GyE)	1.95	1.75	0.54	0.64
SA+AV optimized (GyE)	0.76	0.28	0.89	0.38
SA D_mean_ Δ_relative_	−61.30%	−90.37%	−14.61%	−65.34%
Original (GyE)	14.16	12.15	1.78	6.29
SA+AV optimized (GyE)	5.48	1.17	1.52	2.18
AV D_mean_ Δ_relative_	−61.33%	−94.27%	19.05%	−25.00%
Original (GyE)	0.75	6.46	0.21	0.16
SA+AV optimized (GyE)	0.29	0.37	0.25	0.12
PTV D_95%_ Δ_relative_	−0.42%	−2.95%	3.19%	−0.30%
Original [GyE]	52.40	46.47	66.16	59.05
SA+AV optimized [GyE]	52.18	45.10	68.27	58.87
CTV D_98%_ Δ_relative_	−0.57%	−5.54%	2.84%	−0.39%
Original (GyE)	54.11	64.76	65.84	66.70
SA+AV optimized (GyE)	53.80	61.17	67.71	66.44
GTV D_98%_ Δ_relative_	−0.64%	−6.46%	1.50%	0.00%
Original (GyE)	60.14	46.89	65.34	66.41
SA+AV optimized (GyE)	59.76	43.86	66.32	66.41
Lung sin D_mean_ Δ_relative_	0.93%	−2.44%	25.00%	0.50%
Original (GyE)	10.64	0.82	0.08	4.01
SA+AV optimized (GyE)	10.74	0.80	0.10	4.03
Lung dx D_mean_ Δ_relative_	−0.44%	−7.49%	2.88%	−0.20%
Original (GyE)	16.13	7.88	26.75	19.93
SA+AV optimized (GyE)	16.06	7.29	27.52	19.89
Heart D_mean_ Δ_relative_	−13.27%	−64.80%	0.00%	−14.58%
Original (GyE)	5.64	4.46	6.68	3.36
SA+AV optimized (GyE)	4.89	1.57	6.68	2.87
Esophagus mean D_mean_ Δ_relative_	−0.17%	−50.00%	3.89%	−0.09%
Original (GyE)	30.04	0.04	34.43	43.03
SA+AV optimized (GyE)	29.99	0.02	35.77	42.99
Atrium dx D_mean_ Δ_relative_	−34.42%	−85.38%	−4.35%	−41.10%
Original (GyE)	14.18	3.90	0.23	7.47
SA+AV optimized (GyE)	9.30	0.57	0.22	4.40
Atrium sin D_mean_ Δ_relative_	−7.20%	−72.46%	4.29%	−2.24%
Original (GyE)	24.69	12.78	36.12	13.39
SA+AV optimized (GyE)	22.91	3.52	37.67	13.09
Ventricle dx D_mean_ Δ_relative_	−4.96%	−50.41%	13.43%	−2.89%
Original (GyE)	3.08	1.21	1.34	50.45
SA+AV optimized (GyE)	2.92	0.60	1.52	48.99
Ventricle sin D_mean_ Δ_relative_	−1.35%	−62.16%	17.07%	−1.75%
Original (GyE)	5.38	2.96	0.41	0.57
SA+AV optimized (GyE)	5.30	1.12	0.48	0.56

Abbreviations: MAX, maximal value; MIN, minimal value; SA, sinoatrial; D2%, dose received by 2% of the volume, that is, maximal dose (D_max_); D_mean_, mean dose; AV, atrioventricular; PTV, planning target volume; CTV, clinical target volume; and GTV, gross tumor volume.

Values in Gray equivalent: 1 GyE represents the physical dose of protons multiplied by a relative biological effectiveness factor 1.1 and should thus have similar biologic effects as 1 Gy of photon dose.

## Results

Within our sample of 32 patients, the average SA node D2% (ie, D_max_) was primarily 27.9 GyE, and the average SA node D_mean_ was 14.2 GyE. The median SA node D2% was 26.5 GyE (6.3-70.7), and for D_mean_, it was 8.6 GyE (1.8-51.4). For the AV node, the average AV node D2% was 2.0, and the average AV node D_mean_ was 0.8 GyE. The median AV node D2% was 0.6 GyE (0.1-17.3), and for D_mean_, it was 0.3 GyE (0.0-6.5). All these values represent the sum of phases I and II. In cases of right-sided tumor localization, the SA node received a substantially higher dose compared to left-sided tumor localization. The average SA node D2% for right-sided tumors was 32.5 GyE, compared to 17.1 GyE for left-sided tumors. Within the entire observed sample, the 3 highest values for SA node D2% were 70.7, 57, and 46.8 GyE, with all 3 patients having right-sided tumor localization.

Optimization was performed for 23 patients with SA node D2% exceeding 5 GyE. Results are presented in Table 2. It is evident that the D2% value for the SA node was successfully reduced by almost two thirds (from 27.93 to 10.55 GyE)—the average reduction was 62% (ie, to 38% of the original value). For illustration, in the patient with the highest observed SA node D2% (70.7 GyE), the dose was reduced to 17.5 GyE. The best reduction in SA node D2% was 87%, and the least successful reduction was 17%.

**Figure 2:**
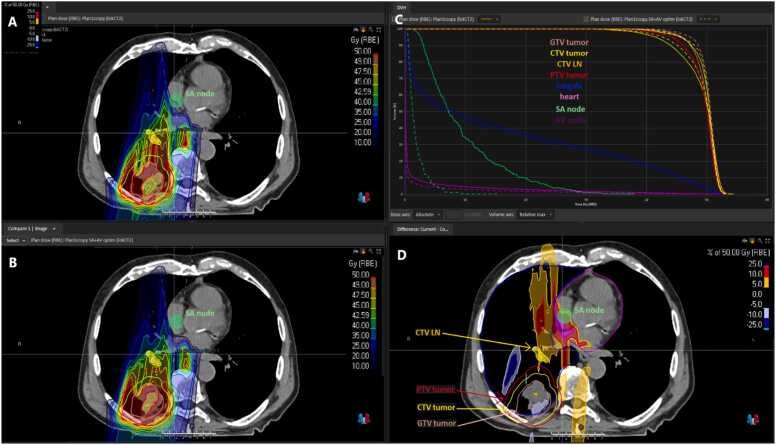
Illustration of dose distribution before and after optimization. Original plan dose distribution (A), SA and AV optimized plan dose distribution (B), DVH comparison (C), and dose difference (D). Abbreviations: AV, atrioventricular; SA, sinoatrial.

Source: Domanský Martin (2023), RayStation Clinical System (12A SP1), Proton Therapy Center Czech, Prague.

We did not observe a significant shift in the dose to other monitored OARs (see Table 2). For example, the average increase in D_mean_ for the left lung was only 0.9%, while for the right lung, there was a reduction of 0.4%. This dose reduction also resulted in a decreased dose to cardiac structures, especially the right atrium. These optimizations led to a reduction in dose exposure to the heart D_mean_ by 13.3% and to the right atrium D_mean_ by 34.4%. Simultaneously, this dose reduction did not result in a substantial decrease in the coverage of PTV, CTV, or GTV. The coverage of target volumes was only negligibly reduced (on average by 0.6%). The reduction in dose to the conduction nodes can be very substantial, with an average reduction of 62.2% for SA node and 61.3% for AV node D2%. The SA and AV nodes D_mean_ were reduced on average by 61.3% both. Illustration of dose distribution before and after optimization see in Figure 2.

## Discussion

The inclusion of heart conduction structures, namely the SA and AV nodes, as OARs is likely to have the greatest significance in diagnoses where cardiotoxicity is most prevalent. This is evident, for instance, in the treatment of lung cancer,^5^, ^6^, ^7^ particularly in cases of right-sided tumors,^20^ mediastinal lymphomas,^8^, ^9^ thymomas,^3^ or esophageal cancer.^21^ The last-mentioned study also illustrates a very high incidence of cardiotoxicity: 21.4% of patients developed incidental AF, and one third developed major adverse cardiovascular events, with the majority of events occurring within ≤2 years of radiation therapy initiation. High doses to the observed cardiac substructures were also associated with worse overall survival.

The risk of developing major adverse cardiac events increases with a rising mean dose (D_mean_) to the entire heart.^7^ However, recent findings suggest that this parameter is not entirely precise, as the heart exhibits significant heterogeneity in terms of radiosensitivity. Across several studies, McWilliam et al^14^, ^15^, ^22^, ^23^ identified the base of the heart as the most radiosensitive region, housing the conduction system or having a close relationship with its origins—the SA and AV nodes. The SA node is located in the myocardium of the right atrium between the opening of the superior vena cava and right atrial appendage on the crista terminalis. It has a curved shape, with dimensions estimated to be around 15 mm in length and a width ranging from 3 to 7 mm.^24^ Impulse is conducted from the SA node through interatrial connections to the left atrium and to the AV node, located in the lower part of the right atrium at the junction of atria and ventricles. The AV node slows down the impulse and transmits it to the bundle of His.

The mean dose (D_mean_) to the entire heart (or V5 and V30) is a routinely monitored parameter but has limitations concerning cardiotoxicity, particularly arrhythmogenic cardiotoxicity.^14^, ^15^, ^22^, ^23^ This is associated with the aforementioned heterogeneity of the heart. Currently, there is no consensus on constraint values for the SA and AV nodes.^13^ At the same time, it remains a question whether the average dose (D_mean_) or maximum dose (D_max_) of the nodes have a more informative value in this case. Few studies have explored the impact of cardiac dosimetry on potentially critical substructures associated with arrhythmias, such as the SA or AV node. However, there are works that investigate the relationship between dose to the conduction structures and subsequent development of arrhythmogenic toxicity. For instance, Chen et al^25^ describe newly developed arrhythmias (mostly supraventricular in origin) after radiation therapy for NSCLC in 7 patients with SA node D_max_ values ranging from 32 to 69 Gy and with SA node D_mean_ values from 17 to 68 Gy. Qian et al^11^ reported the development of symptomatic sick sinus syndrome in a patient with SA node D_max_ of 44.8 Gy.

A retrospective study by Kim et al^10^ proposed a specific constraint value, namely a cut-off value for D_max_ on the SA node. The study evaluated a group of patients with NSCLC and small cell lung cancer after chemoradiotherapy. For patients with small cell lung cancer, exceeding the cut-off value of 53.5 Gy for SA node D_max_ significantly increased the incidence of atrial fibrillation and mortality. In NSCLC, this cut-off value was set even more than twice as low—at 20.0 Gy. The value of 53.5 Gy, proposed by Kim et al,^10^ was exceeded in nearly half of the patients (*n* = 14, 44%) in our dosimetric study on NSCLC patients with N3 disease. In our center so far, we have focused on the whole heart, valves, coronary arteries, and other routinely monitored OARs. According to our findings, the dose to the SA node is high under these optimization procedures. Therefore, including SA node as a new OAR and attempting to reduce the dose will likely be justified, especially for NSCLC (particularly right-sided). Thus, in our dosimetric study, a subgroup was selected from the original 32 patients where the SA node D2% (ie, D_max_) exceeded 5 GyE, for whom we subsequently performed dose optimization. According to our findings, dose reduction to the SA and AV nodes for pencil beam scanning (PBS) is a relatively simple task. This requirement can be met without significantly compromising the quality of the treatment plan in terms of PTV, CTV, or GTV coverage, for which there may often even be an improvement. Other OARs are not negatively affected when including the SA and AV nodes in the optimization process in the case of PBS. The reduction in dose to the conduction nodes can be very substantial.

In dosimetric study on 30 patients with Hodgkin's lymphoma, Loap et al^13^ compared volumetric-modulated arc therapy (VMAT) using photons to IMPT. The results indicated a dosimetric advantage of proton radiation therapy, with significantly lower doses observed for the SA and AV nodes using IMPT compared to VMAT. It should be noted that the conduction nodes were not included among the OARs. Therefore, it is hypothetically possible that, if included, low doses could be achieved using VMAT. However, due to its physical nature, proton radiation therapy allows for easier modulation of the dose distribution, making it easier to avoid exceeding constraints for specific OARs, including the SA and AV nodes. Yet, it remains uncertain whether this dosimetric advantage translates into any clinical benefit. Only a few studies have investigated the correlation between doses to the conduction nodes and a higher incidence of arrhythmias or mortality.^10^, ^11^ Clear confirmation of this hypothesis will require prospective validation with long-term follow-up.

Similarly, as mentioned before, there are no recommended dose constraints for the conduction system. Acquiring these constraints will necessitate more data and studies encompassing the SA and AV nodes as OARs to develop normal tissue control probability models for the conduction system.^13^

To the best of our knowledge, this study represents the first report on optimization directed at the conduction nodes. Previous publications have merely compared measured doses for individual techniques without attempting to re-optimize radiation plans. Our study, for the first time, demonstrates that by incorporating the SA node into the optimization process during IMPT for lung cancer, a substantial reduction in dose to the SA node D_max_ can be achieved without compromising the irradiation of the target volume or increasing doses to target organs.

## Conclusions

In our dosimetric study, we observed relatively high doses to the SA node in a sample of 32 patients with NSCLC with N3 disease. Nearly half of the patients exceeded the threshold of 20 GyE for SA node D2% (ie, D_max_). Optimization with PBS proton therapy resulted in a significant dose reduction (average D2% value for the SA node reduced to almost one third) without compromising the coverage of target volumes or affecting the exposure to OARs. In our opinion, the SA and AV nodes should be considered standard OARs in radiation therapy. Based on our observations with PBS, including the conduction nodes among OARs does not result in a negative impact on other OAR or the coverage of target volumes. Therefore, incorporating conduction structures, especially the SA node, into optimization makes sense for radiation therapy in the thoracic region. Conversely, doses to the AV node were low, approaching 0, for the overwhelming majority of patients. At our center, we now routinely optimize for doses above 5 cobalt gray equivalent, with a priority on D2%, while preserving coverage of target volumes.

Observations from our study are limited by the small number of patients and the narrow specification of the studied diagnosis—NSCLC with N3 disease. Our dosimetric study is solely based on retrospective replanning. Further clarification of the clinical benefit of reducing the dose to the SA and AV nodes is necessary. Prospective studies with a controlled cohort of patients who underwent radiation therapy without dose reduction and a cohort of patients who completely skipped radiation therapy are recommended for this purpose. Additionally, our work explores the possibility of dose reduction only within proton radiation therapy using PBS. The potential for dose reduction to the conduction structures with other radiotherapeutic modalities, especially intensity-modulated radiation therapy and VMAT, must continue to be adequately elucidated.

## Ethics

This study was approved by the Ethics Committee of the institution where this work was conducted.

## Author Contributions

Martin Domanský: Conceptualization, Investigation, Methodology, Writing- Original draft. Michal Andrlík: Formal analysis, Visualisation, Data curation, Methodology. Samuel Kurucz: Formal analysis. Jan Vilimovský: Formal analysis, Data curation. Sarah Al-Hamami Salih and Daniela Šimánková: Investigation. Jiří Kubeš: Conceptualization, Supervision, Project administration, Writing- Review and editing.

## Declaration of Conflicts of Interest

The authors have no conflicts to disclose.

## Declaration of Generative AI and AI-assisted technologies in the writing process

During the preparation of this work, the author used ChatGPT 3.5 in order to check grammar and spelling. After using this tool, the author reviewed and edited the content as needed and takes full responsibility for the content of the publication.

## Funding and Support

This research did not receive any specific grant from funding agencies in the public, commercial, or not-for-profit sectors.

## Data Availability

Research data are stored in an institutional repository and will be shared upon request to the corresponding author.

## References

[1] Ellahham S., Khalouf A., Elkhazendar M., Dababo N., Manla Y. (2022). An overview of radiation-induced heart disease. Radiat Oncol J.

[2] Lyon A.R., López-Fernández T., Couch L.S., ESC Scientific Document Group (2022). 2022 ESC Guidelines on cardio-oncology developed in collaboration with the European Hematology Association (EHA), the European Society for Therapeutic Radiology and Oncology (ESTRO) and the International Cardio-Oncology Society (IC-OS). Eur Heart J.

[3] Taunk N.K., Haffty B.G., Kostis J.B., Goyal S. (2015). Radiation-induced heart disease: pathologic abnormalities and putative mechanisms. Front Oncol.

[4] Yusuf S.W., Sami S., Daher I.N. (2011). Radiation-induced heart disease: a clinical update. Cardiol Res Pract.

[5] Yegya-Raman N., Wang K., Kim S. (2018). Dosimetric predictors of symptomatic cardiac events after conventional-dose chemoradiation therapy for inoperable NSCLC. J Thorac Oncol.

[6] Jang B.S., Cha M.J., Kim H.J. (2020). Heart substructural dosimetric parameters and risk of cardiac events after definitive chemoradiotherapy for stage III non-small cell lung cancer. Radiother Oncol.

[7] Atkins K.M., Rawal B., Chaunzwa T.L. (2019). Cardiac radiation dose, cardiac disease, and mortality in patients with lung cancer. J Am Coll Cardiol.

[8] Jaworski C., Mariani J.A., Wheeler G., Kaye D.M. (2013). Cardiac complications of thoracic irradiation. J Am Coll Cardiol.

[9] Adams M.J., Lipshultz S.E., Schwartz C., Fajardo L.F., Coen V., Constine L.S. (2003). Radiation-associated cardiovascular disease: manifestations and management. Semin Radiat Oncol.

[10] Kim K.H., Oh J., Yang G. (2022). Association of sinoatrial node radiation dose with atrial fibrillation and mortality in patients with lung cancer. JAMA Oncol.

[11] Qian Y., Zhu H., Pollom E.L. (2017). Sinoatrial node toxicity after stereotactic ablative radiation therapy to lung tumors. Pract Radiat Oncol.

[12] Nakao T., Kanaya H., Namura M. (1990). Complete atrioventricular block following radiation therapy for malignant thymoma. Jpn J Med.

[13] Loap P., Mirandola A., De Marzi L. (2022). Cardiac conduction system exposure with modern radiotherapy techniques for mediastinal Hodgkin lymphoma irradiation. Acta Oncol.

[14] McWilliam A., Khalifa J., Vasquez Osorio E. (2020). Novel methodology to investigate the effect of radiation dose to heart substructures on overall survival. Int J Radiat Oncol Biol Phys.

[15] McWilliam A., Abravan A., Banfill K., Faivre-Finn C., van Herk M. (2023). Demystifying the results of RTOG 0617: identification of dose sensitive cardiac subregions associated with overall survival. J Thorac Oncol.

[16] Salim N., Popodko A., Tumanova K., Stolbovoy A., Lagkueva I., Ragimov V. (2023). Cardiac dose in the treatment of synchronous bilateral breast cancer patients between three different radiotherapy techniques (VMAT, IMRT, and 3D CRT). Discov Oncol.

[17] Loap P., Servois V., Dhonneur G., Kirov K., Fourquet A., Kirova Y. (2021). A radiation therapy contouring atlas for cardiac conduction node delineation. Pract Radiat Oncol.

[18] Hattu D., Emans D., van der Stoep J., Canters R., van Loon J., De Ruysscher D. (2023). Comparison of photon intensity modulated, hybrid and volumetric modulated arc radiation treatment techniques in locally advanced non-small cell lung cancer. Phys Imaging Radiat Oncol.

[19] Loap P., Goudjil F., Servois V., Kirov K., Fourquet A., Kirova Y. (2023). Radiation exposure of cardiac conduction nodes during breast proton therapy. Int J Part Ther.

[20] McWilliam A., Vasquez Osorio E., Faivre-Finn C. (2019). Influence of tumour laterality on patient survival in nonsmall cell lung cancer after radiotherapy. Radiother Oncol.

[21] Miller E.D., Wu T., McKinley G. (2024). Incident atrial fibrillation and survival outcomes in esophageal cancer following radiotherapy. Int J Radiat Oncol Biol Phys.

[22] McWilliam A., Kennedy J., Hodgson C., Vasquez Osorio E., Faivre-Finn C., van Herk M. (2017). Radiation dose to heart base linked with poorer survival in lung cancer patients. Eur J Cancer.

[23] McWilliam A., Dootson C., Graham L., Banfill K., Abravan A., van Herk M. (2020). Dose surface maps of the heart can identify regions associated with worse survival for lung cancer patients treated with radiotherapy. Phys Imaging Radiat Oncol.

[24] Csepe T.A., Zhao J., Hansen B.J. (2016). Human sinoatrial node structure: 3D microanatomy of sinoatrial conduction pathways. Prog Biophys Mol Biol.

[25] Chen V., Song A., Werner-Wasik M. (2019). Effect of radiation dose to cardiac substructures on the acute development of new arrhythmias following conventionally fractionated radiation treatment to the lung. Int J Radiat Oncol Biol Phys.

